# Face masks increase compliance with physical distancing recommendations during the COVID-19 pandemic

**DOI:** 10.1007/s40881-021-00108-6

**Published:** 2021-11-13

**Authors:** Gyula Seres, Anna Helen Balleyer, Nicola Cerutti, Anastasia Danilov, Jana Friedrichsen, Yiming Liu, Müge Süer

**Affiliations:** 1 Humboldt-Universität zu Berlin, Spandauer Strasse 1, Berlin, 10178 Germany; 2 University of Groningen, Groningen, CP 9712 The Netherlands; 3 Mercator Research Institute on Global Commons and Climate Change, Torgauer Str. 19, Berlin, 10829 Germany; 4 WZB Berlin Social Science Center, Reichpietschufer 50, Berlin, 10785 Germany; 5 Free University of Berlin, Boltzmannstraße 20, Berlin, 14195 Germany; 6 Einstein Center Digital Future, Wilhelmstraße 67, Berlin, 10117 Germany; 7 DIW, Mohrenstrasse 58, Berlin, 10117 Germany

**Keywords:** COVID-19, Health policy, Face masks, Risk compensation, Social signaling, Field experiment, JEL Classification, C93, D9, I12

## Abstract

Governments across the world have implemented restrictive policies to slow the spread of COVID-19. Recommended face mask use has been a controversially discussed policy, among others, due to potential adverse effects on physical distancing. Using a randomized field experiment (*N* = 300), we show that individuals kept a significantly larger distance from someone wearing a face mask than from an unmasked person during the early days of the pandemic. According to an additional survey experiment (*N* = 456) conducted at the time, masked individuals were not perceived as being more infectious than unmasked ones, but they were believed to prefer more distancing. This result suggests that wearing a mask served as a social signal that led others to increase the distance they kept. Our findings provide evidence against the claim that mask use creates a false sense of security that would negatively affect physical distancing. Furthermore, our results suggest that behavior has informational content that may be affected by policies.

**Supplementary Information:**

The online version contains supplementary material available at 10.1007/s40881-021-00108-6.

## Introduction

Since its first occurrence in late 2019, the coronavirus SARS-CoV-2 had spread to nearly all countries, infected more than 180 million people, and had claimed more than 3.9 million lives by the end of June 2021 (CSSE 2021). As SARS-CoV-2 is most commonly spread via droplets from the mouth or nose, public health authorities recommend regular and thorough hand hygiene, proper coughing and sneezing etiquette, and keeping a safe distance to others (BMG 2020; WHO 2020c). In addition to universally agreed-upon sanitary and social distancing measures, the use of face masks by the general public is a potentially effective but highly debated policy. Not only does the use of face masks by the public vary widely across countries (Belot et al. 2020; IPSOS 2020) but so do official recommendations (Feng et al. 2020). On April 6, the World Health Organization advised that “The use of medical masks in the community may create a false sense of security, with neglect of other essential measures, such as hand hygiene practices and physical distancing...” (WHO 2020b). Despite these claims, by the end of April 2020, many countries, including all German federal states, had made the use of face masks mandatory in stores and public transport. In the same spirit, the Center for Disease Control and Prevention in the US recommends covering one’s face in public where keeping a safe distance is not feasible (CDC 2020). On the other hand, Danish and Norwegian authorities, among others, were decidedly not recommending the use of face masks for healthy people until the second regional wave of the pandemic (Danish Health Authority 2020; Iversen et al. 2020; Danish Health Authority 2021; Vestrheim et al. 2020). The World Health Organization adjusted its position during the outbreak, by May 2020 acknowledging that masks can limit the spread of the virus, although their use alone offers insufficient protection (WEF 2020; WHO 2020a). Despite the contradicting policies, the face mask debate lacks evidence on how mask wearing is perceived by people and how it affects social distancing.

The argument for the community use of face masks is based on studies that found masks to effectively reduce the spread of pathogens when they are worn by infected individuals or universally (van der Sande et al. 2008; Rengasamy et al. 2010; Suess et al. 2012; Saunders-Hastings et al. 2017; Leung et al. 2020; Mitze et al. 2020. Using this evidence, statistical simulations have shown that the universal wearing of a face mask is an effective preventive tool (Eikenberry et al. 2020) against contagion until sufficient testing capacity or vaccination are available. These results are important as SARS-CoV-2 is transmitted also via aerosol (Bahl et al. 2020; Setti et al. 2020). Additionally, the use of a face mask may act as a social signal that itself has the potential to influence the behavior of others. As suggested by Howard et al. (2021), seeing a mask may serve as a reminder to comply with precautionary measures or reveal information about the mask wearer that induces a behavior change in others.^1^ Moreover, by wearing a face mask, an individual might also send a signal that could then influence the attractiveness of following that same behavior through social image concerns and social learning.^2^ For instance, Karing (2021) provides evidence that enabling social signaling significantly increases child immunization in Sierra Leone, both because it is understood as a positive signal that parents value obtaining and because it encourages social learning about the immunization status of others.

The main argument against compulsory face masks emphasizes potentially counterproductive effects from incorrect use, supply shortages, and a false sense of security (WHO 2020a). While supply shortages have been largely addressed and the improper use of masks can be mitigated with training (Javid et al. 2020), there is little evidence for or against the argument that face masks give individuals a false sense of security that would lead to reduced efforts in other precautionary measures. However, there are good arguments to expect such a behavioral backlash. Indeed, masks protect others from infection, who might, in turn, reduce their own preventive efforts in a form of moral hazard (Zweifel and Manning 2000). Similarly, individuals may engage in risk compensation and react to the reduced infection risk from others wearing masks by taking higher risks themselves (Wilde 1982).^3^ Social influence also has the potential to backfire if masks are understood as sending an undesirable signal, so that individuals might engage in compensatory behaviors to send a countersignal. Importantly though, mask policies might interact with and change the social signal associated with a face mask.

Social signaling is an important facet of mask wearing, because individual risk taking has been shown to be sensitive to social influence in other settings. In an abstract experiment, Chung et al. (2015) find that subjects who observed more cautious behavior in others were more likely to choose the safe instead of a risky option themselves. Furthermore, in a study with adolescents, Osmont et al. (2021) find that information on peer behavior is particularly effective when objective information on riskiness is lacking, a feature that describes well the state of information about risk from COVID-19 in spring 2020, when our experiment was run. Seeing someone wear a mask can then be interpreted as informative about the appropriate level of precautions, inducing individuals to keep larger distances toward a masked person. Social influence and signaling require differences in perception and risk taking that were present at the time. Studies from the early phase of the COVID-19 pandemic find that compliance with social distancing mandates varies with perceived risk and that individuals differ substantially in their risk perceptions (Ajzenman et al. 2020; Allcott et al. 2020; Grossman et al. 2020; Harper et al. 2020; Larsen et al. 2020; Rosenfeld et al. 2020; Wise et al. 2020). In contrast to perceived risk, objective risk or social preferences appear to have little effect on (non-)compliance (Canning et al. 2020; Sheth and Wright 2020; Harper et al. 2020). However, due to a potential bias toward socially desirable behaviors (Krumpal 2013; Larsen et al. 2020) or anchoring on widely endorsed behavioral recommendations regarding the safe distance (Kahneman 2011), it is uncertain to which extent survey studies reflect actual behavior.

Given the possibility of behavioral backlash from the universal adoption of face masks during times of high infection rates, it is important to understand how individuals adjust their behavior to masking and how they interpret it (Greenhalgh et al. 2020). To this end, we contribute to the scientific debate on face mask policies from a behavioral perspective. Specifically, we study the effect of masking on physical distancing with a combination of a randomized field experiment and a complementing online survey to examine (1) whether individuals keep a shorter distance to someone who wears a mask and (2) what are the potential reasons behind this behavior. In doing so, we are particularly interested in various aspects of social influence and social signaling. Specifically, we focus on three possible mechanisms. First, wearing a mask can be perceived as a sign of being sick or infectious, because authorities recommend that symptomatic individuals wear masks (ECDC 2020; WHO 2020b). If people knowingly sick with a respiratory disease are more inclined to wear a mask to protect others, a mask becomes a signal for infectiousness and thus encourages further distancing as a precaution. However, if masks signal infectiousness or sickness, wearing a mask becomes less desirable for healthy individuals who would likely prefer not to be perceived as virus carriers. Second, wearing a mask can be perceived as a sign of awareness or anxiety toward the pandemic.^4^ People more concerned about the virus might prefer other individuals to keep a greater distance. Hence, staying further away would be a sign of respect for others’ preferences or reflect a tendency to conform to social expectations (Bernheim 1994; Cialdini and Goldstein 2004). Third, a mask can also serve as a signal about the severity of the ongoing pandemic and one’s share in fighting it. As individuals update their prior on what constitutes appropriate behavior also based on what they see in others and have a tendency to follow perceived norms of behavior, masks can foster compliance with public health rules. Indeed, a study from Japan finds conformity to the social norm to be the most important driver of mask wearing (Nakayachi et al. 2020).

To the best of our knowledge, no evidence supporting the risk compensation argument in the context of face mask wearing is found by various studies run in different countries and different setups (Marchiori 2020; Bakhit et al. 2021; Guenther et al. 2021; Liebst et al. 2021), with the exception of Yan et al. (2020) who argue that mandatory masking caused US Americans to spend more time outside their homes. Our experiment provides the first evidence regarding the effect of mask wearing on social distancing in lines in front of stores, where there is evidence of infection (Qian et al. 2021). Mask use was recommended, but not mandated, by authorities at the time of measurement, making a randomized controlled trial possible. Furthermore, we provide additional insights about distancing with a corresponding online survey experiment in which we elicited subjects’ perceptions on mask use and distancing using pictures of the experimenters with outfits and face masks identical to those in the field study. This allows us to discuss the relevance of various aspects of social signaling in the adoption of precautionary behaviors like mask use and physical distancing.

## Field experiment

In our first study—a randomized field experiment with *N* = 300 conducted in Berlin during the first German lockdown—we tested whether people kept a different distance from individuals with or without a mask when waiting outside a business.^5^ Before arriving at the study site, experimenters wore a mask (Treatment Mask) or not (Treatment NoMask) based on a coin toss. The experimenter took then the last position in a waiting line outside a store, supermarket, or post office. As the next customer arrived and took a position in the line after him, the experimenter measured the distance between themselves and the new customer. The measurement was taken with a light detection and ranging app on a mobile device.^6^ None of the stores had delineated distance markings for the lines. After the measurement was completed, the experimenter moved out of the line, stepped away, recorded the observation, and then returned to the end of the line. The study was conducted in Berlin, Germany, between April 18 and April 24, 2020, before wearing a face mask became mandatory in stores. All data were collected in 21 locations by five experimenters, who acquired 60 independent observations each, divided in 6–11 sessions each (average=8.2), and balanced across the two treatments.^7^ The details of the experimental procedures can be found in the supplementary materials (S3).

Comparing the age groups of the sample from our field experiment to the city’s population shows that the 60+ group is underrepresented (10.7% vs. 24.7%). A likely reason is that seniors contracting the virus were known to face higher death rates and were therefore less likely to take the risk of going out.^8^ However, our sample is meant to represent the relevant population leaving their homes at the time. Furthermore, as we did not observe any age-related effect on distancing, we believe that our observations represent population characteristics well.

On average, subjects kept a distance of 157.2 cm from the experimenter, thus slightly exceeding the mandated minimum distance of 150 cm (*z*=3.565, $\textit{P}\!<$0.01, *n*=300, 2-sided Wilcoxon signed-rank test). However, individual distances varied substantially, ranging from 55 to 275 cm (*SD*=33.3 cm). In the sample, only 61% of the individuals complied with the mandate and stood at least 150 cm away from the experimenter.

**Fig. 1:**
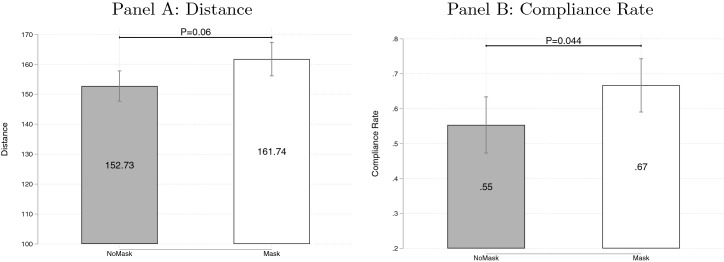
Effect of Mask on Distancing. *Notes:* Panel A shows the average distance kept by subjects in the field experiment in NoMask and Mask treatments. Panel B shows the compliance rate. Bars represent standard errors. *P* values report the results of a Wilcoxon signed-rank test (**A**), *n*=34, and $\chi ^2$ test (**B**), *n*=300

As shown in Fig. 1, the average distance that individuals kept from the experimenter and the compliance rate with the distancing mandate of 150 cm are both significantly higher in the treatment Mask than in the NoMask condition. The average distance is 5.9% or 9 cm larger in the condition where the experimenter was wearing a mask (161.7 cm vs. 152.7 cm, Pr$(|T| > |t|)$ = 0.06, *n*=34, two-sided matched-pair Wilcoxon signed-rank test with two observations per session), and non-parametric kernel density estimates confirm a positive shift in distancing ($\textit{D}$=0.1933, $\textit{P}<$0.01, *n*=300, two-sided Kolmogorov–Smirnov test). Table 1 reports the estimated coefficients of different regression specifications with the distance to the experimenter as the dependent variable. In general, we observe a significantly positive effect of the Mask treatment on the distance ($\textit{P}<$0.10). The statistically significant increase in average distance in the Mask treatment suggests that the argument of masks inducing a false sense of security does not apply when individuals approach a masked person.^9^

Around 17% of the subjects were wearing a mask themselves. If wearing a mask provides a false sense of security, then masks worn by other people should be less relevant to them. Similarly, if masks serve as a reminder or a signal of an elevated subjective risk assessment, people wearing masks are more likely to be alerted to the pandemic. Therefore, we would expect them to react less to the treatment variation. To the contrary, model (4) of Table 1 reveals that the effect of the experimenter having a mask is somewhat stronger but not significantly different for subjects who wore a mask themselves compared to subjects who did not wear a mask. Thus, we do not find any evidence of moral hazard or risk compensation effects of face masks. This result may not extrapolate to a situation with mandatory masking, as subjects wearing a mask voluntarily may differ from the rest of the sample in unobserved dimensions that could influence the distance they keep from others. Moreover, being a self-selected sample, we cannot postulate a causal effect of an individual’s mask wearing on distance keeping.

We further note that subjects who were in the company of other adults came closer to the experimenter than those who were alone. A possible reason is that adult company reduces the attention paid to maintain safe distances from others, because they are, e.g., talking to each other. However, another explanation could be that individuals who are likely to violate the physical distancing rule also take the social distancing rules less seriously and are more likely to be in public places together with others.^10^ Our data do not allow us to distinguish between these factors.

Finally, we address two potential concerns about the experimental design. First, had the subjects been aware of the experiment, that could have influenced their behavior. Although this concern cannot be refuted with certainty, it is highly improbable that a subject could notice the measurement taking place. As the experimental protocol explains, the measurement of distance only took seconds and the experimenter recorded additional information on each observation only after leaving the waiting line and before joining it again. Second, the treatment—wearing a mask—could have deterred an individual from joining the line. We argue that a subject turning away from a line they wanted to join as a consequence of the experimenter wearing (or not wearing) a face mask would increase the time between observations, decreasing the length of the waiting lines. By testing the sample correlation coefficient between the number of people in the waiting line and the treatment variable, this is rejected (*r* = − 0.0722, *p* = 0.212, *n* = 300).^11^

**Table 1:** Treatment effect on physical distancing

Dependent variable: Distance in cm	(1)	(2)	(3)	(4)
Mask Experimenter	8.519$$^{**}$$	8.566$$^{**}$$	9.476$$^{**}$$	7.450$$^{*}$$
	(3.757)	(4.062)	(4.037)	(4.308)
Mask Subject	14.83$$^{***}$$	13.48$$^{***}$$	10.77$$^{***}$$	4.436
	(4.904)	(4.392)	(3.758)	(5.816)
Mask Experimenter $\times$ Mask Subject				11.93
				(12.58)
Accompanying Adult			-12.02$^{*}$	-11.44$^{*}$
			(5.217)	(5.340)
Accompanying Child			2.610	2.686
			(5.464)	(5.519)
Gender of Subject			3.156	3.305
			(3.063)	(3.126)
Population Density of Neighborhood			-0.00122	-0.00123
			(0.000911)	(0.000916)
Length of the Line			1.219$$^{***}$$	1.232$$^{***}$$
			(0.338)	(0.338)
Constant	150.5$$^{***}$$	155.5$$^{***}$$	168.2$$^{***}$$	168.5$$^{***}$$
	(2.717)	(7.639)	(13.65)	(13.74)
Control Variables	No	Yes	Yes	Yes
Observations	300	300	300	300
$R^{2}$	0.046	0.135	0.190	0.194

*Notes:* Ordinary least-squares estimates. Robust standard errors in parentheses. $$^{*}$$ $$p<0.10$$, $$^{**}$$ $$p<0.05$$, $$^{***}$$ $$p<0.01$$. Mask Experimenter and Mask Subject are indicator variables for whether the experimenter or subject, respectively, used a face mask. Gender=1 if the subject is female. Accompanying Adult and Accompanying Child indicate whether the subject was accompanied by at least one other adult or child, respectively. Population density is based on the 2011 German Census data. Length of the Line indicates the number of people in front of the shop. Control variables are age groups, store types, and experimenter fixed effects

## Survey experiment

Having found a positive causal effect of face masks on physical distancing, we next investigate potential explanations for this result in a survey experiment with *N* = 456. The survey was conducted online with individuals living in Germany via www.prolific.co on April 26, 2020, before federal face-covering mandates came into force in Germany.

First, each respondent was randomly exposed to a photograph of an original experimenter from the field experiment either wearing a face mask (Mask treatment) or not (NoMask treatment).^12^ We also randomized the person in the picture, to closely mimic the setting of our field experiment. Then, respondents were asked to imagine the pictured person to be queuing in a waiting line outside of a post office and estimate (i) the distance to this person at which another individual joining the line would come to stand (in cm); (ii) the distance the pictured person would prefer the arriving individual to keep from him or her (in cm), and (iii) how likely it is that the pictured person is sick, or (iv) infectious (both answers were given on a 7-point Likert scale). Next, respondents were asked to guess the average answers of 50 other randomly selected survey respondents [(in case of (i) and (ii)], or modal answers [(in case of (iii) and (iv)]. We rewarded each correct guess with a bonus of 0.20 EUR. Finally, the respondents were asked to estimate which distance 30 participants of a past field experiment had kept from the pictured person on average (in cm). Again, we rewarded this estimate with a bonus of 0.20 EUR if it was within a range of 5 cm of the actual distance. Thus, one key feature of our framework is that respondents were not only asked their opinion about the possible behavior, but also had to predict the answers of other respondents to the same questions, as well as the actual behavior in the field experiment. Table S6 in the online supplementary material S2.2 reports means and standard deviations of outcome variables, and tests of treatment differences corrected for multiple hypothesis testing (List et al. 2019).

At the end of the survey, we collected the respondents’ attitudes regarding mask mandates, hygiene rules compliance, and demographic characteristics. Table S3 in the online supplementary material S2.2 provides an overview of the distribution of the respondents between treatments regarding their demographic characteristics.

**Fig. 2:**
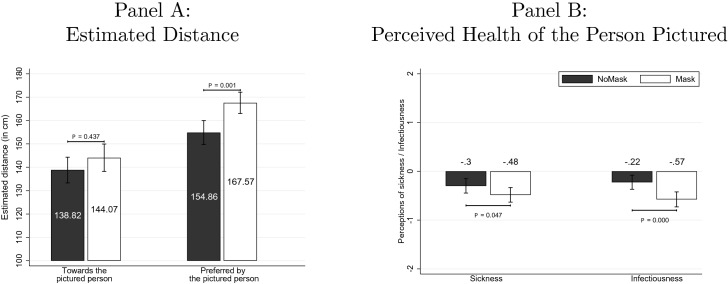
Testing channels with survey respondents. *Notes:* The left panel pictures the estimated average distance kept in the field experiment and beliefs about the average perception of other respondents about the preferred distance in treatments Mask and NoMask. The right panel illustrates the chances of the person pictured being sick or infectious in treatments Mask and NoMask. -3 stands for “definitely not sick” or “definitely not infectious“, 0 stands for “I’m not sure”, and 3 stands for “definitely sick” or “definitely infectious’.’ The $\textit{P}$ values are based on the results of the two-sided Mann–Whitney *U* test. All values in Panel B are significantly different from zero ($\textit{P}<$0.05, *n*=456, two-sided Wilcoxon signed-rank test.)

Figure 2 summarizes the main results of the survey. Respondents predicted that the average distance toward the masked experimenter in the field experiment would not be shorter than the one to the unmasked experimenter (144.07 cm vs. 138.82 cm, *z* = − 0.777, *P* = 0.437, *n* = 456, two-sided Mann–Whitney *U* test). We derive this result from the incentivized question on guessing the average distance kept in the field experiment, but the results are consistent if we use the hypothetical question about the distance the next person in line would keep (151.50 cm in Mask vs. 144.87 cm in NoMask, *z* = − 1.423, *P* = 0.155, *n* = 456, two-sided Mann–Whitney *U* test). Thus, the survey respondents recognized that wearing a face mask does not drive shorter distances to the masked person, but they underestimated the mask’s positive effect on the distance kept. This observation also suggests that the mask is not interpreted as a signal of a riskier situation, which is in line with the observation from the field experiment that also masked subjects react to the treatment.

Next, we examine whether the mask is interpreted as informative about the mask wearer himself or herself. To that regard, we investigate two different signals that the mask might send. First, it might signal that the wearer is more anxious or risk averse and, therefore, prefers larger distances. Second, it might signal that the wearer is infectious or sick, wearing the mask to protect others. Both signals could be a reason why subjects kept larger distances from the masked than the unmasked experimenter.

We first investigate whether (i) people who wear masks are perceived as those who prefer to keep larger distances from others and whether (ii) this perception actually results in longer distances. To test (i), we compare across treatments the respondents’ perception of the preferred distance that the person on the picture would like others to keep from him or her. Respondents in Mask stated that they believe the pictured person with a face mask to prefer a distance of 166.14 cm, on average. In NoMask, the average answer to the same question was 148.29 cm. The treatment difference is statistically significant (*z* = − 4.394, $P\!<$0.01, *n* = 456, two-sided Mann–Whitney *U* test). A similar picture also arises when we look at the second-order beliefs regarding the preferred distance. In our context, the second-order beliefs are beliefs about the average (or mode, in case of questions with the Likert scale) answer of 50 other randomly selected survey respondents to the respective question. The second-order beliefs about the preferred distance of the pictured person are, on average, higher in the treatment with Mask (167.57 cm) than in NoMask (154.86 cm). The treatment difference is statistically significant ($\textit{z}$= − 3.205, $\textit{P}\!<$0.01, *n* = 456, two-sided Mann–Whitney *U* test). This finding suggests that wearing a mask is perceived by the survey participants as informative about the wearer. Wearing a mask thus serves as a social signal that separates those who prefer longer distances from those who tolerate shorter ones. Our data do not allow to further probe whether it is anxiety, risk attitudes, or something else that is driving this preference.

**Fig. 3:**
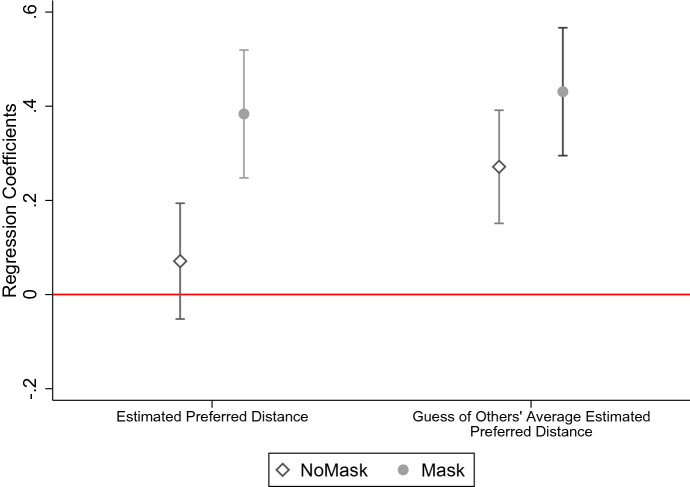
Responsiveness of the respondents’ guesses of the average distance kept in the field experiment to the expected preferred distance. *Notes:* This figure plots coefficients obtained from an ordinary least-squares regression of the survey respondents’ estimate of the average distance kept by subjects in our field experiment on their first- and second-order belief about the preferred distance of the experimenter in both Mask and NoMask conditions and the respective 95% confidence intervals. The control variables used in the regressions are the respondents’ perception of the sickness/infectiousness of the pictured person, levels of compliance with lockdown measures in the past week, beliefs toward the effectiveness of masks, and demographic information including age, gender, income, household size, political views, and risk attitude. See Table S7 in the Supplementary Materials for the detailed estimation results

To examine (ii), whether the signal also affects the behavior of others, we next test whether the *estimate of the average distance kept by the participants of the field experiment* can be predicted by the respondent’s first- or second-order beliefs about the *preferred distance of the pictured experimenter*. Figure 3 provides a graphical illustration of the regression results reported in Table S7 in the online supplementary material S2.2. All coefficients are positive and most of them are significant. We note that the first-order beliefs about the *preferred distance* are not significantly correlated with the *estimated average distance in the field experiment* in NoMask once we include controls. However, this correlation is highly significant and positive in Mask (0.384, $P\!<$0.01), and the difference between the two coefficients is significantly different from 0 at the 5% level. Moreover, the second-order beliefs are significantly and positively correlated with the *estimated distance in the field experiment* in both treatments. Also, in this case, the correlation is higher in Mask, even though the difference is not significantly different from 0. In summary, these findings suggest that the respondents who expect other individuals to believe that the pictured person prefers longer distances from other people also believe that other individuals actually kept a longer distance from the pictured person. The relationship is present, albeit weaker, also for the first-order beliefs about the preferred distance and the guess of the average distance in the field experiment. In other words, those respondents who perceive the mask as signaling a preference for larger distances also expect others to react to this information and increase their distance to the experimenter in response.

The fact that the correlation between beliefs about the preferred distance and the guess of actual distances kept is larger in Mask can be interpreted as the respondents believing that the masks make it easier for the subjects to guess the correct distance. One potential reason could be that the mask makes people think of the actual rules of containment, so that estimated preferred distances, the guessed and the actual distances, are anchored with the recommended distance of 150 cm in Mask, whereas no anchor exists in NoMask.

We also investigate an alternative channel for social signaling. As masks mostly protect others from getting infected (masks are primarily seen as an instrument of source control according to Howard et al. , 2021), one might suspect that wearing a mask is interpreted by others as a sign of infectiousness. However, we fail to find evidence that at this time during the pandemic, people perceived a masked person as more likely to be sick or infectious than a person without a mask (Fig. 2 Panel B). To the contrary, experimenters in Mask were perceived as less likely to be sick (*z* = 1.981, $\textit{P}$= 0.0475, *n*=456, two-sided Mann–Whitney *U* test) and as less likely to be infectious (*z* = 3.631, $\textit{P}\!<$0.01, *n* = 456). Therefore, we rule out that the mask serves as a sign of someone being sick or infectious and therefore motivates other people to stay further away to avoid infections. This finding is intuitive in the context of the pandemic as individuals with any sign of an infection or a known contact to an infected person were asked to strictly stay at home and thus should not be expected in public waiting lines.

Altogether, our data suggest that wearing a mask serves as a social signal and significantly affects the behavior of others, triggering greater distancing. Interestingly, the effect is not driven by an increased perception of risk from a potentially infectious or sick mask wearer (mask signaling individual needs to wear a mask). Instead, the result is rather driven by a positive signaling mechanism, where masks are taken as indicative of the wearers’ desire for greater distancing, which then indeed affects distancing.

## Discussion

While policymakers ponder how to best protect public health, the universal use of face masks (i.e., also by healthy individuals) is a prominently discussed option and has been implemented at some phase of the pandemic in many countries. Using data from Germany (Mitze et al. 2020), the US (Chernozhukov et al. 2021), and Canada (Karaivanov et al. 2021), researchers find that the introduction of mandatory masking has substantially reduced the spread of the virus and the cumulative death count and provides a cost-effective instrument to curb virus spread. However, despite their widespread use and these positive findings at the aggregate level, little is known as to how masks affect individuals’ behaviors and perceptions. We argue that we need to better understand mask wearing and its behavioral effect to best design non-pharmaceutical interventions that help navigate the long-term endeavor of life with SARS-CoV-2 or other future threats to public health.

This study contributes to the understanding of face masks as a tool for curbing the spread of COVID-19 by providing evidence on the effect of face masks on distances kept by others as well as additional evidence on potential drivers behind the observed effect that include social signaling as a prominent and potentially powerful driver of precautionary behaviors. Specifically, we developed a field experiment to test whether the use of face masks affects compliance with the public health mandate of keeping a sufficient physical distance from others. Using a randomized treatment design, we measured the distance maintained by individuals from an experimenter in a public line waiting to enter a business.

In our sample, we find robust evidence that face masks increase distancing. If the experimenter was wearing a face mask, subjects stood on average 9 cm further away than if the experimenter was unmasked. The compliance rate with the distancing mandate of 150 cm increased by more than 10 percentage points from 55% to 67%. We further find that subjects wearing a mask themselves keep a larger distance from the experimenter, whereas individuals in groups keep a significantly shorter distance. Using a complementary survey experiment, we show that masked individuals are not perceived as more likely to be sick or infectious. However, they are believed to prefer to keep a larger distance from others, which our respondents expect subjects in the field experiment to respect.

Our results contradict the hypothesis of risk compensation that predicts individuals to reduce their precautions toward masked as compared to unmasked individuals. However, our findings are consistent with the idea that masks signal to others that they should adopt stricter precautions, because (a) the situation is severe or (b) they interpret the mask wearer as someone preferring greater caution. Using the various aspects of beliefs from the survey experiment, we argue that it is only (b) that drives the result as we find no evidence that respondents who saw a masked experimenter adjusted their perceptions on the usefulness of masks in various settings upward as compared to those seeing the unmasked experimenter.

Our findings have important implications for the discussion of face coverings. First, our study suggests that individuals do not let down their guard when someone else is wearing a mask. On the contrary, masks foster efforts to comply with the recommendation of physical distancing.^13^ Second, our probing into the mechanisms suggests that social signaling is an important aspect in this area. Mask wearing is perceived as informative about individuals’ preferences and possibly decisions (we did not elicit the latter).^14^ This suggests that encouraging voluntary mask use through information campaigns may be even more effective in inducing behavior change than mask mandates and may under certain circumstances be preferable. This is because mask mandates can only signal the perceived severity at the aggregate level. At the same time, however, a mandate weakens the information sent through the individual use of a mask simply because it is not a voluntary choice anymore. Moreover, if a mandate is instituted at the state level and not locally, also the signal about the severity of the situation is weakened as compared to a situation with endogenous mask use.^15^ Voluntary masking, in contrast, gives full potential to beneficial social learning and signaling effects that might be particularly important in a situation like the early days of the pandemic, where little was known about the virus and its risks. We expect that these effects could be further increased by information campaigns featuring well-connected individuals or influencers.

We further acknowledge that mask mandates additionally run the risk of politicizing the issue of whether or not to wear a mask, because they have to be instituted in the political arena. The decision to wear a mask can then become associated with signals entirely unrelated to their beneficial public health aspects. In the worst case, political signals associated with mask use may lead to the negative effects of social influence in the respective political camp. Information campaigns are, in our view, less likely to be controversial. However, as noted by Betsch et al. (2020), if opinions on the necessity of masks are already divided and possibly so along preexisting policy cleavages, a mandatory policy might be more advisable to reduce the risk of increased polarization and stigmatization.

We would like to point out that our study provides insights only into part of the issues at hand. To fully assess the effects of mask wearing, one would also want to understand the behavioral effects on the wearer themselves. A different study design would be required to do so. The challenge we see is that randomization of who wears a mask raises ethical concerns as not wearing a mask may be associated with a health risk that the respective individuals would not want to bear. This concern does not apply to the experimenters in this study who have no known risk factors and decided to undertake the study under strict compliance with recommendations by the local health authorities.

## Supplementary Information

Below is the link to the electronic supplementary material.

Supplementary file1 (PDF 321 KB)

## Data Availability

All data and code for replication will be made available to researchers for purposes of reproducing or extending the analysis upon publication of the study.

## Footnotes

1 This argument is in line with the original idea of Spence (1973) that, in a separating equilibrium, by choosing certain actions, individuals can credibly signal their type and will then be treated differently by others depending on their signal.

2 Social influence often runs through several channels. In the context of financial investment decisions, Bursztyn et al. (2014) find that peer effects in financial decisions are driven both by a social learning effect and by a desire to “keep up with the Joneses”, i.e., a social image effect. See also Bénabou and Tirole (2006) for a seminal contribution on image concerns in prosocial behavior and Bursztyn and Jensen (2017) for a general discussion of social pressure in the field.

3 Evidence on risk compensatory behavior in the context of HIV prevention (Eaton and Kalichman 2007; Marcus et al. 2013; Wilson et al. 2014), seat-belt laws (Evans and Graham 1991; Cohen and Einav 2003; Houston and Richardson 2007), and bicycle helmets (Adams and Hillman 2001) is mixed. Based on a theoretical model of risk compensation by Blomquist (1986), Seres et al. (2021) argue that risk compensation may fail in the context of COVID-19, because masks may make complying with other measures easier, contradicting the model.

4 Nakayachi et al. (2020) provide evidence that one important reason for mask wearing in a Japanese sample is the desire to reduce own anxiety.

5 Before collecting the data, we did not have a specific hypothesis regarding the effect of wearing a mask on distances. See the preregistration of the field experiment for details: 10.1257/rct.5735-1.0.

6 The measurement was not obvious to the subjects, took 5–20 s, and we did not record any instance when they were clearly aware of it.

7 For more information on the sessions and the locations, please refer to S1.2 in the online supplementary material.

8 A large number of local neighborhood initiatives offered free shopping services for the elderly and for people at high risk from COVID-19.

9 We account for heteroscedasticity created by locations and date of measurement and for the relatively small number of clusters. In models 2, 3, and 4, our observations are clustered by shop and date of measurement. To account for the limited number of clusters, we use a wild-t cluster bootstrap as in Cameron et al. (2008), using the implementation proposed by Roodman et al. (2019). Table 1 reports the p values obtained with the wild-t bootstrap procedure.

10 At the time of the experiment, citizens of Berlin were asked to avoid any unnecessary social contact with individuals from other households.

11 As we use a between-subject design, an order effect may play no role in subjects’ distancing behavior. However, a measuring error may occur if the precision of the experimenter’s measurement is influenced by the order. Including the starting treatment as a dummy variable in the fully specified Model (4) in Table 1, we reject this hypothesis (*p* = 0.109).

12 Original photographs are available upon request.

13 A field experiment conducted in Italy reports similar results, suggesting that our findings are not pertaining to Germany only (Marchiori 2020). Our design allows straightforward replications of the experiment in other environments and countries.

14 The signal that is extracted from someone wearing a mask will likely depend on the type of mask and who is wearing it as well as on the legal and political stance on masking. Our experiment featured young people. We expect that the effect might be even larger when older individuals who are more at risk from infection wear masks. On the contrary, the effect might be smaller when someone is seen with an incorrectly fitted mask or a mask offering less protection to the wearer than the FFP2 masks used in our study. Such questions of heterogeneity go beyond this paper.

15 Evaluating the effects of various mask mandates in the US, Seegert et al. (2020) argue that the introduction of county-level mask mandates led households to infer an increased risk of infection that in turn made them increase their precautions, because authorities would only introduce a mandate if the risk was high. However, they do not find the same effect for state-level mandates, arguably because these are less informative as to the local risk.
