# Supplementary material for: Face masks increase compliance with physical distancing recommendations during the COVID-19 pandemic
Source: J Econ Sci Assoc. 2021 Nov 13;7(2):139–58. doi: 10.1007/s40881-021-00108-6 (PMC8590132; doi:10.1007/s40881-021-00108-6)
Supplement: Supplementary file 1 — Supplementary file1 (PDF 321 KB) [file 40881_2021_108_MOESM1_ESM.pdf]

Face masks increase compliance with physical distancing recommendations during the COVID-19 pandemic

Supplementary Material

by Gyula Seres, Anna H Balleyer, Nicola Cerutti, Anastasia Danilov, Jana Friedrichsen, Yiming Liu, and Müge Süer

The first supplementary material is a collection of sections describing the field experiment with greater detail and includes technical details, supplementary descriptive statistics, and a randomization check. The second unit contains details of the online survey experiment with similar content. The third unit is the protocol for the field experiment. The last section is an English translation of the text of the survey experiment as well as the complete original survey in German including both treatment conditions with one of the experimenters.[16] The IRB approval is attached to the end of this file.

[16] Pictures of the other confederates are available on request.

Appendix S1 Study 1 - Field Experiment

S1.1 Experimental procedures

Throughout data collection, the use of face masks was recommended by the Berlin state government but not mandated.[17] Businesses typically regulated how many customers were allowed to enter their premises at the same time to ensure compliance with the physical distancing mandate. At the time, in Berlin people were required by a state directive to keep a 150 cm distance to non-household members in public spaces.[18] During the period of data collection, the regulatory circumstances did not change.

During data collection, experimenters followed a predefined dress-code and an experimental protocol (see Section S3 for details). Each experimenter collected data in public lines of people waiting to enter a store, supermarket, or post office. Data was collected in daylight to ensure good visibility and on flat surfaces to allow for precise measurements. At the beginning of each data collection, the experimenter determined via a coin toss whether to start with Mask or NoMask. They would switch to the other treatment after a predetermined number of observations and collect an equal number of observations in both treatments.

In the treatment condition, Mask, only FFP2-type face masks were used.[19] We measured and recorded the distance between the arriving next person and the experimenter (see Section S3 for details on the procedure).[20] We estimate the effect of masks on distancing as the difference between the average recorded distances in (Mask) and (NoMask) treatments.

To start data collection, the experimenters took a position at the end of the line, ensuring a distance of 150 cm to the person in front of them, assuming a sideways position in the line. When the next person arrived (the subject), the experimenters recorded the distance between their own and the subject's feet.[21] The experimenter proceeded to the next observation by returning to the end of the line until the predetermined number of observations was reached.

---

[17] Mandatory use of masks was first introduced in some public spaces in multiple steps starting from April 27, 2020 (Berlin Senate, 2020). Note: The announcement was made after the end of the data collection for the field experiment.

[18] In Germany, most policies were within the discretion of the individual states but the federal government and talks between state governments lead to largely uniform rules. In Berlin, the policies to limit the spread of COVID-19 including physical distancing were regulated through the SARS-CoV-2 Containment Measures Ordinance (SARS-CoV-2-EindmaßnV) on March 22, 2020; the ordinance was changed several times since but not in a respect relevant to the experiment (Berlin Senate, 2020).

[19] An FFP2 face mask or filtering facepiece respirator is a half-face mask that filters the air inhaled by the wearer. Details are specified in the EN 149 standard, an equivalent of the N95 US standard. At the time of data collection, the device was the most commonly available protective mask. It is identical to the one used in the online survey.

[20] FFP2 respirators must meet high filter standards and are among the most effective commercially available face coverings. However, at the time of the field experiment, no government policy was in place encouraging the community use of masks, nor any information campaign informing the public about mask types. Moreover, there was an acute shortage of masks, and surgical masks were in general unavailable. Hence, we do not believe that the mask choice played a role in the measurements at the time.

[21] The measurement was recorded by an augmented reality application on a mobile device that is able to measure a distance between two points on a flat surface in 1-centimeter increments. To comply with privacy laws, no visual recording was taken.

A distance was not recorded if the target subject changed position during the measurement or when the camera view was obstructed by, for example, a signpost. When a group approached the end of the line, the distance was measured to the person standing closest to the experimenter. If the closest person was an infant in a stroller or a person in a wheelchair, the point used for measurement was where the front wheel touched the ground.[22]

All data was collected in Berlin, Germany, between April 18 and April 24, 2020, by five experimenters, aged 31 to 35, two women and three men, who acquired 60 observations each, balanced across the two treatments.[23]

S1.2 Experimental results

Descriptive statistics and randomization checks

Our sample consists of independent observations from 300 subjects, 48.7% of whom were male. The majority of subjects were estimated to be between 25 and 45 years old (58.3%). The percentage of subjects entering the line alone was 80.4%, whereas 12.6% were accompanied by at least one adult and 7% were with at least one child. At the time of measurement, 17% of the subjects were wearing a face mask.

Table S1: Randomization check for the field experiment

	Overall (N = 300)	NoMask (N = 150)	Mask (N = 150)	Significant difference between conditions
Male	49%	49%	48%	χ^2 = 0.053, P = 0.545 [a]
14 and under	1%	1%	1%	
Aged between 14 and 25	10%	8%	13%	
Aged between 25 and 35	33%	33%	32%	z = -0.421, P = 0.674 [b]
Aged between 35 and 45	26%	28%	23%	
Aged between 45 and 60	20%	21%	20%	
Aged 60 and older	10%	9%	11%	
Mask Subject	17%	15%	19%	χ^2 = 0.591, P = 0.269 [a]
Company Adult	13%	12%	13%	χ^2 = 0.121, P = 0.431 [a]
Company Child	7%	7%	7%	χ^2 = 0.051, P = 0.500 [a]
Length of line	7.0 (5.2)	7.4 (5.6)	6.6 (4.8)	t = 1.249 P = 0.106 [c]

Notes: The reported statistics are based on: [a] 1-sided Pearson's Chi-square-Test [b] 2-sided Mann-Whitney U-Test [c] 1-sided T-test. Values in brackets are standard deviations.

S1.3 Kernel density estimates

Using non-parametric kernel density functions, we estimate the distribution of the distance values separately in the two treatments (Fig. S1). A positive

[22]Dogs were not included in the study as SARS-CoV-2 has been shown to replicate poorly in canines (Shi et al, 2020).

[23]All experimenters participated in data collection voluntarily and are credited as co-authors of this article. None of the authors were in an employee-employer relationship, mitigating ethical concerns that might arise because time spent in public for data collection during the pandemic may pose a certain health hazard.

Table S2: Summary information on the field experiment's sessions

Session	Date	Shop id	Time	Experimenter	n	Store	Start
1	18apr	11	14:29	1	9	Drug store	NoMask
2	18apr	6	19:28	4	6	Supermarket	NoMask
3	18apr	2	11:15	5	10	Post office	Mask
4	19apr	16	13:42	3	6	Coffee shop	NoMask
5	20apr	11	17:30	1	6	Drug store	Mask
6	20apr	12	12:50	1	16	Post office	NoMask
7	20apr	13	16:28	1	10	Drug store	NoMask
8	20apr	15	16:43	2	6	Convenience store	Mask
9	20apr	18	14:20	2	11	Post office	NoMask
10	20apr	1	16:27	3	8	Post office	Mask
11	20apr	6	18:46	4	8	Supermarket	NoMask
12	20apr	7	13:14	4	12	Post office	NoMask
13	20apr	8	12:34	4	6	Bakery	NoMask
14	20apr	2	11:15	5	14	Post office	NoMask
15	19apr	16	14:00	3	8	Coffee shop	NoMask
16	21apr	5	11:29	2	12	Post office	NoMask
17	19apr	16	13:29	3	6	Coffee shop	NoMask
18	21apr	17	13:12	2	8	Food stall	NoMask
19	21apr	1	15:57	3	6	Post office	Mask
20	21apr	7	16:00	4	12	Post office	NoMask
21	21apr	20	14:15	5	9	Post office	Mask
22	22apr	3	18:10	2	4	Supermarket	NoMask
23	22apr	17	19:10	2	8	Food stall	Mask
24	20apr	7	13:28	4	2	Post office	Mask
25	22apr	1	11:56	3	10	Post office	Mask
26	22apr	14	12:52	3	9	Post office	NoMask
27	22apr	6	15:40	4	10	Supermarket	NoMask
28	21apr	7	16:06	4	2	Post office	NoMask
29	22apr	9	11:55	4	2	Phone repair shop	Mask
30	20apr	2	11:15	5	3	Post office	Mask
31	22apr	2	10:30	5	17	Post office	NoMask
32	23apr	12	12:16	1	18	Post office	Mask
33	23apr	4	11:41	2	2	Bakery	Mask
34	23apr	10	12:33	2	3	Supermarket	Mask
35	23apr	21	11:05	2	4	Coffee shop	NoMask
36	23apr	14	15:00	3	5	Post office	NoMask
37	24apr	11	12:25	1	1	Drug store	NoMask
38	24apr	19	11:35	2	2	Post office	NoMask
39	24apr	1	11:36	3	2	Post office	NoMask
40	24apr	2	11:15	5	5	Post office	Mask
41	20apr	2	11:15	5	2	Post office	NoMask

Notes: Summary information for each session on date, location, starting time, experimenter running the session, number of observations, store type and starting treatment.

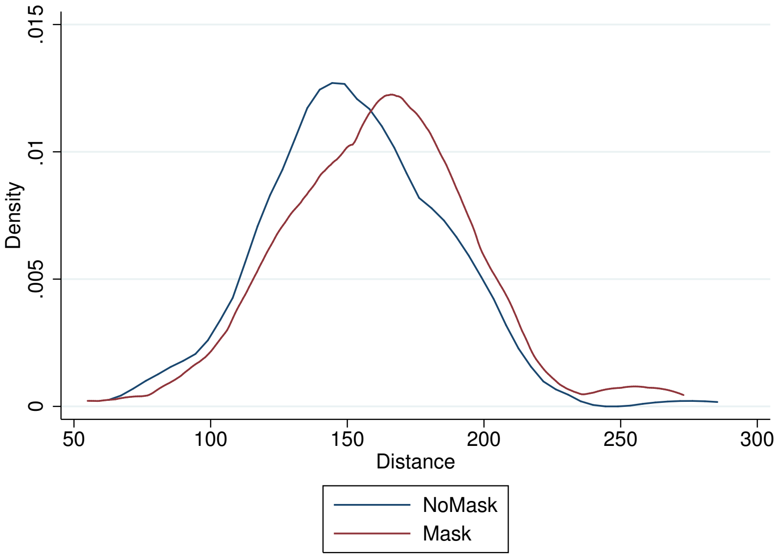

Fig. S1: **Kernel density estimates of subject distance.** Estimated univariate Epanechnikov kernel density functions of distance maintained by the subject from the experimenter. The two graphs are calculated separately using the NoMask and Mask treatments.

shift in distancing can be statistically confirmed (D=0.1933, P <0.01, 2-sided Kolmogorov–Smirnov test) and it demonstrates that the presence of a mask induces individuals to keep a greater distance.

Appendix S2 Study 2 - Survey Experiment

S2.1 Survey design and procedures

The survey was conducted via www.prolific.co. The subject pool was restricted to adult individuals who live in Germany (see Table S4 for the geographical distribution). The survey language was German. The translation of questions can be found below in Section S4). In total, the sample consisted of 463 observations; 7 observations were excluded from the analysis due to having failed attention checks (about the gender, pose, mask, and hair color of the pictured person) leading to a final sample of 456 used for the analysis. The survey lasted on average 8.5 minutes.

The survey participants were paid 2.15 EUR for their participation. An additional bonus was paid for some questions. On average, the bonus amounted to 0.18 EUR. All payments were made via the website of the subject pool provider www.prolific.co.

A key feature of our framework is, that respondents were not only asked their opinion about the possible behavior but also had to predict the most popular answers of other respondents to the same questions. For each correct prediction, the respondents received a bonus of 0.20 EUR.

S2.2 Descriptive statistics and randomization checks

Table S3 reports the average descriptive statistics by treatment. As can be seen, the respondents' characteristics are equally distributed between treatments with a small exception regarding respondents' native language: the share of the German native speakers is by 6.1 percentage points lower in the NoMask condition. This difference is at the 10% significant (two-sided t-test) but not significant when tested with Mann-Whitney U test (z = -1, P = 0.3173). Another difference in the sample between treatments is that the respondents in the NoMask condition report to have taken part in a slightly larger number of studies about the masks than those in the Mask treatments (t = 1.65, P = 0.0995). The results of the Mann-Whitney-U test are not significant (z = 1.552, P = 0.1207).

The average age of respondents in the sample is 28.1 (SD = 8.2) years. Of the respondents, 58.77% are male, 8.77% of respondents identified themselves as belonging to the risk group for COVID-19, and a further 2.4% answered they were not sure. Virtually all respondents live in Germany. Respondents' distribution by German federal states is reported in Table S4 and largely corresponds to the distribution of the German population.

The average household size of the respondents is 2.6 (SD = 1.82) persons. The income distribution for the subsample of respondents who provided an answer to the question about their household income is given in Table S5.

Respondents also reported their past compliance with recommended prevention measures. Average compliance on a 6-point Likert scale ranging from 1 'never' to 6 'always' was for hand-washing 4.7 (SD = 1.08), for wearing a face mask indoors 2.2 (SD = 1.38), for wearing a mask outdoors 2.1 (SD = 1.42), and for keeping a 150 cm distance to people they do not share a household with 5.0 (SD = 0.94).

The survey further elicited attitudes toward possible mask mandates using a 5-point Likert scale ranging from -2 for 'very negative' to 2 for 'very positive.' A mandate for wearing a mask in supermarkets and public transport was evaluated positively (M = 1.21, SD = 0.94 and M = 1.17, SD = 0.94, both P = 0, 2-sided Wilcoxon signed-rank test). However, a possible mandate to wear a mask while walking outside was perceived negatively (M = -0.49, SD = 1.21, P = 0). On average, the respondents indicated that they perceived face masks as being relatively effective in preventing the spread of the coronavirus (M = 0.78, SD = 0.92, P = 0).

Table S3: Demographic characteristics and attitudes towards COVID-19 measures

Variable	(1) NoMask	(2) Mask	(3) NoMask vs. Mask
Age	27.952	28.325	0.373
	(8.560)	(7.828)	(0.628)
Male	0.571	0.615	0.044
	(0.496)	(0.488)	(0.339)
Live in Germany	0.996	1.000	0.004
	(0.066)	(0.000)	(0.318)
Household size	2.583	2.601	0.018
	(2.156)	(1.396)	(0.918)
Mother-tongue German	0.789	0.851	0.061*
	(0.409)	(0.357)	(0.088)
General risk attitude	4.855	4.895	0.039
	(2.005)	(2.056)	(0.836)
COVID-19 risk group	0.110	0.114	0.004
	(0.313)	(0.319)	(0.882)
Survey experience about masks	1.075	0.833	-0.241*
	(1.700)	(1.407)	(0.100)
Survey experience about COVID	4.921	4.544	-0.377
	(2.719)	(2.595)	(0.130)
Perceived face masks effectiveness (general)	0.789	0.772	-0.018
	(0.924)	(0.915)	(0.839)
Perceived face masks effectiveness for walking outside	-0.575	-0.408	0.167
	(1.153)	(1.261)	(0.142)
Perceived face masks effectiveness in public transport	1.189	1.158	-0.031
	(0.907)	(0.972)	(0.727)
Perceived face masks effectiveness in supermarkets	1.215	1.206	-0.009
	(0.897)	(1.014)	(0.922)
Frequency of compliance with distance of 150 cm	5.004	4.961	-0.044
	(0.922)	(0.959)	(0.619)
Frequency of compliance with hand-wash for 20 sec.	4.719	4.671	-0.048
	(1.050)	(1.111)	(0.634)
Frequency of compliance with wearing masks outdoor	2.009	2.145	0.136
	(1.405)	(1.436)	(0.307)
Frequency of compliance with wearing masks indoor	2.189	2.197	0.009
	(1.371)	(1.389)	(0.946)
Observations	228	228	456

Notes: Column 1 and Column 2 report mean answers in NoMask and Mask conditions respectively. Standard deviations in parentheses. Column 3 reports the difference between the treatments. The significance levels of two-sided t-test are reported on superscripts. * $p < 0.10$, ** $p < 0.05$, *** $p < 0.01$.

S2.3 Additional results

Table S6 reports the mean and standard deviations for our key outcome variables. Column 3 reports the differences between treatments and the p-values for the multiple hypothesis testing as described by List et al (2019).

Table S4: Origin of respondents in the online survey by German federal states (in %)

State	Survey sample	Population in Germany in 2018
North Rhine-Westphalia	21.9	21.6
Bavaria	16.7	9.6
Lower Saxony	12.3	15.8
Baden-Württemberg	10.3	13.3
Berlin	9.2	4.4
Hessen	7	7.6
Saxony	4.4	4.9
Rhineland-Palatinate	3.7	4.9
Brandenburg	2.6	3
Hamburg	2.4	2.2
Bremen	2.2	0.8
Schleswig-Holstein	2	3.5
Mecklenburg-Vorpommern	1.8	1.9
Thuringia	1.5	2.6
Saarland	1.3	1.2
Saxony-Anhalt	0.7	2.7

Notes: Column 2 reports the distribution of the respondents' location over the federal states in the survey sample. Column 3 shows the distribution of German population in 2018 over the federal states according to the Federal Statistic Office, https://www-genesis.destatis.de/genesis/online Code "12411-0010".

Table S5: Distribution of the household income in the survey sample (in%)

Income brackets	Survey sample	German households in 2018
less than 1,500	31.2	25.7
1,500 - 2,000	15	15.4
2,000 - 2,600	15.5	15.7
2,600 - 3,200	12.1	11.7
3,200 - 4,500	10.9	16.6
4,500 - 6,000	9.2	8.7
more than 6,000	6.1	6.2

Notes: The distribution of the household income of the respondents in the survey sample is reported in column 2. Column 3 shows the distribution of the income of German households in 2018 according to the Federal Statistic Office, https://www-genesis.destatis.de/genesis/online Code "12211-0105."

Table S6: Outcome variables and results of multiple hypothesis testing

Variable	(1)<br>NoMask	(2)<br>Mask	(3)<br>NoMask vs. Mask
Belief about av. distance kept by the participants	138.816	144.075	5.259
of the field experiment	(42.116)	(44.887)	(0.337)
Belief about preferred distance	148.285	166.145	17.860***
of the pictured person	(38.491)	(36.437)	(0.000)
Second-order belief about other respondents'	154.864	167.570	12.706***
belief about the preferred distance	(39.171)	(34.908)	(0.000)
Belief about distance kept	144.873	151.500	6.627
by a person behind	(39.536)	(41.185)	(0.247)
Second-order belief about other respondents'	151.215	152.118	0.904
belief about the distance kept by a person behind	(39.644)	(37.638)	(0.805)
Estimated likelihood of pictured	-0.404	-0.825	-0.421***
person being infectious	(0.857)	(0.950)	(0.000)
Mode answer of other respondents about	-0.224	-0.575	-0.351***
the likelihood of infectiousness	(1.110)	(1.179)	(0.002)
Estimated likelihood of pictured	-0.500	-0.728	-0.228**
person being sick	(0.883)	(0.883)	(0.03)
Mode answer of other respondents about	-0.298	-0.482	-0.184
the likelihood of sickness	(1.130)	(1.148)	(0.232)
Observations	228	228	456

Notes: Column 1 and Column 2 report mean answers by treatment and standard deviations in parentheses. Column 3 reports the difference between the treatments. P-values for multiple hypotheses testing (List et al, 2019) are reported in parentheses. * $p < 0.10$, ** $p < 0.05$, *** $p < 0.01$.

Table S7: Responsiveness of estimated distance to first- and second-order beliefs about the pictured experimenter

Dependent Variable: Estimated distance in the field experiment				
Panel A: First Order Beliefs				
	(1)	(2)	(3)	(4)
First-order belief about preferred distance	0.224**	0.445***	0.071	0.384***
	(0.072)	(0.076)	(0.074)	(0.082)
First-order belief about sickness	-1.486	4.647	-0.426	5.378
	(3.839)	(3.750)	(3.798)	(3.882)
First-order belief about infectiousness	-2.287	-6.563	-0.588	-7.641*
	(3.972)	(3.484)	(3.976)	(3.543)
Subsample	NoMask	Mask	NoMask	Mask
Control variables	No	No	Yes	Yes
Observations	228	228	226	226
R-squared	0.050	0.147	0.308	0.293
Panel B: Second Order Beliefs				
	(1)	(2)	(3)	(4)
Second-order belief about preferred distance	0.386***	0.441***	0.284***	0.403***
	(0.069)	(0.081)	(0.072)	(0.084)
Second-order belief about sickness	1.174	-1.659	0.954	-1.565
	(3.137)	(3.642)	(3.098)	(3.845)
Second-order belief about infectiousness	3.594	3.743	2.614	3.545
	(3.186)	(3.525)	(3.164)	(3.690)
Subsample	NoMask	Mask	NoMask	Mask
Control variables	No	No	Yes	Yes
Observations	228	228	226	226
R-squared	0.123	0.126	0.357	0.284

Notes: Ordinary least squares estimates. Standard errors in parentheses. * $p < 0.10$, ** $p < 0.05$, *** $p < 0.01$. This table shows detailed estimation results obtained from a linear regression of survey respondents' *estimated average distance kept by the participants of the field experiment* on their first- and second-order beliefs about the experimenter in Mask and NoMask. In both panels, we consider three types of beliefs: beliefs about the pictured person's preferred distance, beliefs about the likelihood of the person being sick, and beliefs about the likelihood of the person being infectious. In panel A, the independent variables are all three first-order beliefs. In panel B, we instead use second-order beliefs, which are beliefs about the average or mode answer of other respondents about the preferred distance, the sickness and the infectiousness. The first-order beliefs are not incentivized, but the second-order beliefs are incentivized. In models (3) and (4), the control variables are levels of compliance with lock-down measures in the past week, beliefs toward the effectiveness of masks, and demographic information consisting of age, gender, income, household size, political view, and risk attitude. Excluding the believes about the sickness and infectiousness of the pictured person does not change the results.

Appendix S3 Protocol for the field experiment

Disclaimer: *The experimenters who collected data in the field experiment signed up to do so voluntarily and confirmed that they did not belong to any higher risk group according the classification of the Robert Koch Institute.*[24] *In order to prevent imposing health risks on others, the RKI's health recommendations were strictly followed at all times of the experiment.*
Given the particular circumstances in the early days of the pandemic for the field experiment and that HU Berlin had no ethics committee at the time, we requested and received an ex post IRB approval from the dean of research. The approval is available at the end of the Supplementary Material.

Introduction

The instructions for recording the data follow. Please read the whole document and follow all points very carefully.

Code of Conduct

Experimenter Appearance

As an experimenter, you will need an FFP2 respiratory protection mask for this experiment. **Each time before** you go to an experiment location, you will take two full-body (self-)portrait photos of yourself: One with and one without a mask. The primary purpose of the photos is to record variables describing your appearance if this is requested by the reviewers. To decrease the noise due to experimenter appearance, you are expected to wear a pair of blue jeans and a dark-colored (black, dark gray or navy blue) top without any visible text or logo.[25]

Location You may choose a location that satisfies the following list of conditions.

- The establishment is an open supermarket, a drug store (except pharmacy) or a post office.
- There must be a waiting line outside with people waiting to enter the store. The waiting line must stand on a flat surface with no obstructing objects. Make sure that the waiting line is clearly visible and it is clear for the arriving subject that you are the last person in the line and approximately where they should stand.
- You can record the data anytime until April 24 between 8am to 8pm during daylight with good visibility. In order to secure good visibility conditions, **do not** record data when it is raining.
- You should avoid stores that have heavy traffic that would make measurement difficult. For instance, if there is another store or a subway exit next

[24] The Robert Koch Institute (RKI) is the government's key scientific institution in the field of biomedicine. It is one of the central bodies for the safeguarding of public health in Germany. It identifies risk factors that increase the chance of a serious illness; we confirm that none of the experimenters fall into these categories. See `https://www.rki.de/`.

[25] Please consult us if you do not own these items.

door, people in the waiting line might change their position frequently, making recording data problematic.

- The time gap between people who are let into the store must be sufficiently long. The measurement may take a couple of seconds, and you may be asked to move forward if the waiting line moves; the subject can also move before you can record the distance between you. The speed is usually smaller at post offices than at supermarkets.

Data Recording Method You will need a smartphone with an installed augmented-reality tape-measure app that is capable of measuring small distances in centimeters with small measurement errors. The error is measured individually on the same device you use on location. Place two flat objects on the ground at any location with a clear surface exactly 100 cm from each other. Similarly to the protocol on location, measure this distance with the application. Do the same measurement five times with different positions of the objects. You may proceed with this hardware and application if the error is within a 3% margin every time.

Preparation for Data Recording In total, you are expected to perform 60 independent observations. Before each session, you set an even target of observations you are planning to record. Half of them you execute with, the other half without your mask on. The order you decide randomly using a fair coin or any random number generator. Example: You set the number to 20. After tossing the coin, you start with 10 observations with your mask on. After finishing with this, you remove the mask and perform another 10 without it. Finally, you leave the location.

The purpose of changing your appearance only once is to limit the number of times you may accidentally touch your face. You can safely avoid this if you remove the mask by only touching the strings. You should proceed the same way if you start your work without your mask on. To learn about the safe way to wear a mask, please consult the website of the Robert Koch Institute.

Data Recording Procedure Due to lockdown measures in place, you will work alone and record the data individually. After choosing the location, go to the end of the waiting line outside and carefully follow this protocol.

1. Go to the waiting line and stand 150 centimeters (1.5 meter) away from the last person.[26] Measure the distance using the same application.
2. Turn sideways, not facing either the waiting line nor the subject arriving after you. Make sure that you can see both.
3. If necessary, calibrate your application such that it is ready for measurement. Do not open other applications at this point.
4. If someone approaches, turn your back to the waiting line and face the subject before they arrive. Make sure that your face is visible, but look

[26] Recommended minimum safe distance by the Federal Government of Germany and the Robert Koch Institute.

at your device the whole time. Keep a neutral facial expression and do not make eye contact.
5. The app measures distance by pinning two points on the ground. These two points are the closest points of yours and the subject's shoes. You pin the tip of their shoe first when they arrive, and the tip of your shoe second.
6. Record the length and exit the waiting line.
7. After this, record all remaining variables, starting with the number of people in the waiting line who were standing before you outside at the point of measurement. After this, go back to the end of the waiting line until you reach your target number of observations.

Further Points to Consider

If there is a group, the subject is the person closest to you, irrespective of age. Exceptions: If the closest person is an infant in a stroller or a person in a wheelchair, the closest point is where the front wheel touches the ground. If this reference point belongs to a stroller, the person you record is the one handling the stroller.

Do not record an observation if you are unable to pinpoint the position of the subject accurately (i.e., the subject might keep jogging in place, or move back or forward before you can finish pinning) or if the subject engages in an activity that would trigger distancing according to local social norms (i.e., smoking, talking on the phone, eating).

There are three time slots per day: morning 8am-12 noon, afternoon 12 noon-16pm, and late afternoon/early evening 4pm-8pm. Do not record more than 50% of the observations in one period of time (e.g., morning), even if they are recorded on different days.

Do not attempt to make any media recording of the subject or any other individual near you as without consent this may be unwelcome. If you meet with a hostile or unfriendly reaction or you are questioned by someone, you can reveal your identity and that you are conducting a publicly funded scientific study. If this hinders or influences recording data, or puts you in an uncomfortable situation, leave the location.

Data and Variables

In this part, you can find the list of variables with the corresponding codes. Your task is to complete the spreadsheet for each observation. You will receive the spreadsheet by email. Once you have finished recording, send the file to gyula.seres@hu-berlin.de.

MaskE	Treatment variable. Experimenter 0=without 1=with mask.

Distance	Distance to the subject. Measured in centimeters (cm).
GenderS	Binary variable. Subject gender 0=male 1=female.
AgeS	Guessed age category of the subject. 0= below 14, 1=14-25, 2=25-35, 3=35-45, 4=45-60, 5=60+. If it is uncertain, write your best guess.
MaskS	Binary variable. Subject 0=without 1=with mask.
CompanyAdult	Number of accompanying adults, 0=no adult. Adult, if age>14.
CompanyChild	Number of accompanying children, 0=no child. Child, if age<14.
TotalNumofPeople	The total number of people outside in front of you in the waiting line at the moment of measurement. Do not include people inside.
SocialNormS	The presence of social norm violations (i.e., smoking, food, other).
Address	Address of the experiment. For example, “Spandauer Strasse 1, 10178.”
Store	Type of the store. 1=post office, 2=supermarket, 3=drug store, 4=other (please add a note)
ID	Surname of experimenter.
Date	Date of the month. For example, if the date is April 20, write 20.
Time	Time (i.e., 1400, 1430, etc.).
Note 1	Additional remarks, may be left empty.
Note 2	Additional remarks, may be left empty.

Appendix S4 Survey questionnaire

The original survey was written in German. Below, we provide an English translation. We structure the text with informative subheadings that were not part of the survey text that respondents saw.

Welcome to this study on judgment and decision-making. This survey will take 15 minutes of your time. Every person who completed a survey, including you, will receive 2.15 EUR for participation. The payment will be processed via prolific.co and done automatically. Please read all questions carefully and answer them truthfully.

Introduction of the picture Below, you can see a picture of a person in front of the post office. Please answer the following questions with regard to the picture you see.

- To which extent do you agree,

  ...that the person pictured looks relaxed?

  ...the person pictured looks tidy?

  ...the person pictured looks friendly?

1 “strongly agree” 2 “moderately agree” 3 “ agree a little” 4 “neither agree nor disagree” 5 “disagree a little” 6 “moderately disagree” 7 “strongly disagree”

- Have you seen this person before? Yes / No / Maybe

Opinion about the preferences and the health condition of the person (not) wearing a mask, the effectiveness of masks for distancing

Imagine the following situation: The person you saw in the photograph at the beginning of the survey is standing in a waiting line outside of a post office. Now another person (who is interested in getting into the post office) approaches the end of the waiting line.

- In your opinion, at which distance will the person approaching come to stand behind the person in the photograph. Please indicate the distance in centimeters below (100 cm = 1 m).
- What do you think is the minimum distance the person in the photograph would like the person approaching the waiting line to keep from her/him while waiting in line outside a post office? Please indicate the distance in centimeters below (100 cm = 1m).
- In your opinion, how likely is it that the person in the photograph is infectious for other people in the waiting line? Please choose one answer from 1 to 7. 1 “definitely not infectious” 2 “very unlikely to be infectious” 3 ”somewhat unlikely to be infectious” 4 “I don’t know” 5 “somewhat likely to be infectious” 6 “very likely to be infectious” 7 “definitely infectious.”
- In your opinion, how likely is it that the person pictured is sick with the coronavirus, the flu, or another virus-related respiratory diseases? Please choose one answer from 1 to 7. 1 “definitely not sick” 2 “very unlikely to be sick” 3 ”somewhat unlikely to be sick” 4 “I don’t know” 5 “somewhat likely to be sick” 6 “very likely to be sick” 7 “definitely sick.”

Introduction of the bonus rules

In the upcoming part of the survey you will be able to earn some additional bonus payment. You will be asked to estimate the average or most frequent answers of other survey participants. For each correct guess, you will receive an additional payment of 0.20 EUR (20 cents). More details about the rules for bonus payment will be given below.

Please enter your Participant ID here if you would like to receive the payment. It will be used for payment purposes only. After the payment has been made, it will be deleted from the data set.

Incentivized beliefs / Descriptive social norm elicitation

Other survey participants were shown the same photograph as you at the beginning of the experiment and were asked the same questions as you.

All participants saw the following situation description: “Imagine the following situation: The person you have seen in the photograph at the beginning of the survey is standing in a waiting line outside of a post office. Now another person (who is interested in going into the post office) approaches the end of the waiting line.”

Please estimate the average answers to the following two questions by 50 randomly selected individuals. Think about your answer thoroughly, because for each guess that does not deviate from the actual average answer of 50 other participants by more than 5 cm, you will receive an additional bonus of 0.20 EUR.

- What is the average answer of 50 other randomly selected participants to the following question: “At which distance will the arrived person come to stand behind the person in the photograph.” Please guess the average answer to this question:
- What is the average answer of 50 other randomly selected participants to the following question: “What is the minimum distance this person would like the next person in the waiting line to keep from him/her while waiting in line outside a post-office?.” Please guess the average answer to this question:

Now, we would like you to estimate the most frequent answer among 50 randomly selected participants of this survey. Think about your answer thoroughly, because for each correct guess you will receive a bonus of 0.20 EUR.

- What is the most common answer among 50 randomly selected survey participants to the following question: “How likely is it that the person in the photograph is infectious for other people in the waiting line? (From 1 to 7)” Please guess the most common answer to this question: 1 “definitely not infectious”; 2 “very unlikely to be infectious”; 3 ”somewhat unlikely to be infectious”; 4 “I don’t know”; 5 “somewhat likely to be infectious”; 6 “very likely to be infectious”; 7 “definitely infectious.”
- What is the most common answer among 50 randomly selected survey participants to the following question: “How likely is it that the pictured

person is sick with the coronavirus, the flu, or another virus-related respiratory disease? (From 1 to 7)" Please guess the most common answer to this question: 1 "definitely not sick"; 2 "very unlikely to be sick"; 3 "somewhat unlikely to be sick"; 4 "I don't know"; 5 "somewhat likely to be sick"; 6 "very likely to be sick"; 7 "definitely sick."

Estimation of the experimental results

Last week we ran a study in which we measured the distance that individuals keep at the end of a waiting line from another person. The study was done in Berlin in a line for the post office. The last person in the waiting line was an experimenter, who you saw in the picture at the beginning of the survey.

Please guess the average distance 30 individuals kept from this person.

Think about your answer thoroughly, because you can earn an additional bonus based on the correctness of your guess. If your guess does not deviate from the actual average distance from our study by more than 5 cm, you will receive an additional bonus of 0.20 EUR.

- Please guess the average distance kept away from the experimenter by 30 individuals approaching him/her at the end of the waiting line:

Attitude towards masks and mask-wearing behavior

- How do you evaluate the introduction of the compulsory wearing of face masks in public transport in Germany? 1 "very positive"; 2 "rather positive"; 3 "undecided"; 4 "rather negative"; 5 "very negative."
- How do you evaluate the introduction of compulsory wearing of face masks in supermarkets? 1 "very positive"; 2 "rather positive"; 3 "undecided"; 4 "rather negative"; 5 "very negative."
- How do you evaluate a possible introduction of compulsory wearing of face masks while walking outside? 1 "very positive"; 2 "rather positive"; 3 "undecided"; 4 "rather negative"; 5 "very negative."
- In your opinion, to what extent are face masks effective for preventing the spread of coronavirus? 1 "very effective"; 2 "somewhat effective"; 3 "I don't know"; 4 "not very effective"; 5 "not effective at all."
- In the last week, how often did you : (1 "never" to 6 "always")
  wash hands with soap for at least 20 seconds.
  wear a face mask in indoor areas
  wear a face mask in outdoor spaces
  keep a distance of at least 150 cm to people who are not living in your household.
- There are some groups of people who are at particular risk of developing a serious disease due to infection with the coronavirus. These groups include people who are over 65 years of age, have a weakened immune system, or have a relevant underlying medical condition (e.g., chronic diseases of the respiratory system, diabetes, cardiovascular diseases, cancer). Do you belong to a coronavirus risk group? Yes/No/Maybe.

Past experience with coronavirus-related survey

- How many times have you participated in surveys about COVID-19 / coronavirus in the last 4 weeks? Scale 0 to "10 or more."

- How many times have you taken part in surveys about face masks in the last 4 weeks? Scale 0 to "10 or more."

Attention check

- Does the person you saw at the beginning of the survey wear a mask? Yes/No
- What is this person's hair color? (Multiple choice: Blond/Brown, etc)
- Was this person standing or sitting?
- What is the gender of the pictured person? male/female

Demographic questions

Please answer the following questions about yourself:

- How old are you?
- What is your gender? male/female/diverse
- Do you live in Germany? yes/no
- In which federal state do you live? (Choice from a drop-down menu)
- Are you, in general, a risk-loving or risk-averse person? (1=not risk-loving at all, ..., 10=very risk-loving)
- How many people live in your household (including yourself)?
- What is your average monthly net household income? "Less than 1,500 EUR", "Between 1,500 EUR and 2,000 EUR", "Between 2,000 EUR and 2,600 EUR", "Between 2,600 EUR and 3,200 EUR", "Between 3,200 EUR and 4,500 EUR", "Between 4,500 EUR and 6,000 EUR", "6,000 EUR or more", "I don't want to answer this question"
- Which party would you vote for if the Bundestag elections were on Sunday in Germany? SPD / CDU / CSU / FDP / Bündnis 90 (Die Grünen) / Die Linke / AfD / NPD(Republikaner/Die Rechte) / Other / No answer
- Is German your native language? yes/no

Survey comprehension and comments

- Did you have problems understanding the survey? yes/no
- If so, what exactly were you not clear about? (text box)
- You can leave us a comment or a suggestion here. (text box)

Thank you for your participation, you have reached the end of the survey! Your payment will be processed automatically. If you are eligible for the additional payment, you will be notified within 72 hours.
